# RNA-Binding Protein HuR Suppresses Inflammation and Promotes Extracellular Matrix Homeostasis via NKRF in Intervertebral Disc Degeneration

**DOI:** 10.3389/fcell.2020.611234

**Published:** 2020-11-25

**Authors:** Zhenxuan Shao, Zhuolong Tu, Yifeng Shi, Sunlong Li, Aimin Wu, Yaosen Wu, Naifeng Tian, Liaojun Sun, Zongyou Pan, Linwei Chen, Weiyang Gao, Yifei Zhou, Xiangyang Wang, Xiaolei Zhang

**Affiliations:** ^1^Department of Orthopaedics, The Second Affiliated Hospital and Yuying Children’s Hospital of Wenzhou Medical University, Wenzhou, China; ^2^Key Laboratory of Orthopaedics of Zhejiang Province, Wenzhou, China; ^3^The Second School of Medicine, Wenzhou Medical University, Wenzhou, China; ^4^Department of Burn, The First Affiliated Hospital of Wenzhou Medical University, Wenzhou, China; ^5^Department of Orthopaedics, The Second Affiliated Hospital of Zhejiang University School of Medicine, Hangzhou, China; ^6^Chinese Orthopedic Regenerative Medicine Society, Hangzhou, China

**Keywords:** intervertebral disc degeneration, HuR, inflammation, extracellular matrix, NKRF

## Abstract

Intervertebral disc degeneration (IVDD) has been reported to be a major cause of low back pain. Studies have demonstrated that IVDD may be dysregulated at the transcriptional level; however, whether post-transcriptional regulation is involved is still unknown. The current study aimed to illustrate the role of Human antigen R (HuR), an RNA binding protein involved in post-transcriptional regulation, in IVDD. The results showed that the expression of HuR was decreased in degenerative nucleus pulposus (NP) tissues as well as in TNF-α-treated NP cells. Downregulation of HuR may lead to increased inflammation and extracellular matrix (ECM) degradation in TNF-α-treated NP cells; however, these effects were not reversed in HuR overexpressed NP cells. Inhibition of the NF-κB signaling pathway attenuates inflammation and ECM degradation in HuR-deficient NP cells. A mechanism study showed that HuR prompted NKRF mRNA stability *via* binding to its AU-rich elements, and upregulation of NKRF suppressed inflammation and ECM degradation in HuR-deficient NP cells. Furthermore, we found that NKRF, but not HuR, overexpression ameliorated the process of IVDD in rats *in vivo*. In conclusion, HuR suppressed inflammation and ECM degradation in NP cells *via* stabilizing NKRF and inhibiting the NF-κB signaling pathway; NKRF, but not HuR, may serve as a potential therapeutic target for IVDD.

## Introduction

Low back pain is a common disorder in the population, and up to 25% of people in the United States suffer from back and neck pain (Martin et al., 2008; Hoy et al., 2010; Zheng et al., 2018). Severe low back pain can lead to disability, which not only affects the quality of life for individuals but also causes a serious burden to society; the socio-economic burden caused by low back pain has been reported to exceed 85 billion dollars (Martin et al., 2008). Studies have shown that up to 40% of lower back pain is related to intervertebral disc degeneration (IVDD) (Freemont, 2009).

The intervertebral disc is the soft tissue located between the bony vertebrae, and it absorbs the load transmitted by the vertebral body and provides flexibility for the spine. The intervertebral disc is composed of three parts: cartilage endplate, annulus fibrosus (AF), and nucleus pulposus (NP). The jelly-like NP tissue is the major tissue of intervertebral disc to combat against loading, while the dysfunction of NP cells is considered to be the initiating cause of IVDD (Sakai and Grad, 2015; Gorth et al., 2020).

The function of cells can be regulated at various levels, such as transcriptional and post-transcriptional (Uchida et al., 2019). In the previous studies, we found that transcription factor BRD4 is overexpressed in degenerated NP cells, and it may promote matrix metallopeptidase 13 (MMP13) expression *via* MAPK and NF-κB signaling pathway (Wang et al., 2019). We also showed that the nuclear localization of transcription factor EB (TFEB) was declined in NP cells during IVDD, which caused blockage of autophagy flux and increased apoptosis (Zheng et al., 2019). Also, dysfunction of SOX9 and FOXO has been implicated in IVDD (Gruber et al., 2005; Alvarez-Garcia et al., 2017). These studies reveal that the pathogenesis of IVDD may occur at the transcriptional level; however, whether post-transcriptional is involved is still unclear.

Post-transcriptional regulation refers to the regulation of gene expression at the post-transcriptional level, such as RNA stabilization, RNA cleavage, RNA editing, RNA splicing, and translation (Zhao et al., 2017). RNA-binding protein is a type of post-transcriptional regulation protein, which can bind to RNA and participate in the functional regulation (Glisovic et al., 2008). Human antigen R (HuR), encoded by the ELAVL1 gene, is one of the first identified RNA binding proteins. HuR can bind to the adenine-uracil-rich elements (ARE) at the 3′UTR of the target mRNA molecule and regulates a variety of biological processes (Schultz et al., 2020). Pan et al. (2019) found that HuR may regulate extracellular matrix (ECM) gene expression and pH homeostasis in physiological hypoxia condition in NP cells; however, the role of HuR in pathogenesis of IVDD is still unknown.

In the current study, we evaluated the expression of HuR in NP cells during IVDD; we also upregulated and downregulated its expression *via* lentivirus to explore the role of HuR in IVDD; inflammatory genes were screened to demonstrate its working mechanism; HuR and NKRF overexpressing lentiviruses were injected into NP tissues to assess their therapeutic effect on IVDD.

## Materials and Methods

### Ethics Statement

All surgical interventions, treatments, and postoperative animal care procedures were performed in strict accordance with the Guide for the Care and Use of Laboratory Animals of the National Institutes of Health and the Animal Care and Use Committee of Wenzhou Medical University (WYDW2020-0560). Human tissue collection and treatments were also permitted by the Second Affiliated Hospital and Yuying Children’s Hospital of Wenzhou Medical University Ethics Committee (LCKY2020-135).

Human NP tissues of different grades were isolated from patients, to compare the expression of ELAVL1 by quantitative PCR (qPCR). The work was given official approval by the Ethics Committee of the Second Affiliated Hospital of Wenzhou Medical University, and we obtained written informed consent from patients or relatives prior to tissue collection.

### Reagents and Antibodies

Dimethyl sulfoxide (DMSO), citrate buffer, and collagenase type II were purchased from Sigma-Aldrich (St. Louis, MO, United States). The cell culture reagents were obtained from Gibco (Grand Island, NY, United States). Primary antibodies against IL-1, TNF-α, p65, GAPDH, and Lamin B were obtained from Proteintech (Wuhan, China); primary antibodies against matrix metallopeptidase 3 (MMP3), MMP13, IL-6, Aggrecan, COL2, and NKRF were acquired from Abcam (Cambridge, MA, United States); and primary antibodies against HuR (ELAVL1), p-p65, and IkBa were purchased from Cell Signaling Technology (Beverly, United States). Goat anti-rabbit and anti-mouse IgG-HRP antibodies were purchased from Bioworld (Nanjing, China). Alexa Fluor^®^ 488- and Alexa Fluor^®^ 594-conjugated secondary antibodies were obtained from Jackson ImmunoResearch (West Grove, PA, United States). 4′,6-Diamidino-2-phenylindole (DAPI) was obtained from Beyotime (Shanghai, China).

### Culture of NP Cells and Extraction

Rat NP cells were extracted from healthy NP of young Sprague-Dawley rats. NP tissues were cut up into 1 mm^3^ and washed for three times with phosphate-buffered saline (PBS) before digestion with 0.25% type II collagenase at 37°C for 2 h. After centrifugation, the cell suspensions were cultured in DMEM/F12 (Gibco) with 10% fetal bovine serum (FBS; Gibco) and antibiotics (1% streptomycin/penicillin) in the incubator at 5% CO_2_ at 37°C.

### Quantitative Real-Time PCR (qPCR)

After treatment, total RNA was extracted from NP cells by the TRIzol method (Invitrogen). One thousand nanograms of total RNA was reverse transcribed to synthesize cDNA (MBI Fermentas, Germany) using the PrimeScript-RT reagent kit (Takara, Japan) and the CFX96 Real-Time PCR System (Bio-Rad Laboratories, Berkeley, CA, United States). Amplification of the cDNA was performed by SYBR Premix Ex Taq using the CFX96 Real-Time PCR System (Bio-Rad Laboratories, Berkeley, CA, United States). The cycle threshold (Ct) values were determined and normalized to the level of a housekeeping gene (GAPDH). The expression of the target genes in different groups was evaluated using the 2^–ΔΔCt^ method. The forward and reverse primer sequences are provided in Supplementary Table S1.

### Western Blot (WB) Analysis

Proteins from NP cells were lysed in radioimmunoprecipitation assay (RIPA) buffer (Beyotime) with 1 mM phenylmethanesulfonyl fluoride (PMSF) (Beyotime) and then centrifuged for 20 min at 12,000 rpm at 4°C. The protein concentration was measured by the BCA protein assay kit (Beyotime). Forty nanograms of protein was separated via 8–12% (w/v) sodium dodecyl sulfate polyacrylamide gel electrophoresis and blotted onto polyvinylidene fluoride membranes (Bio-Rad, Hercules, CA, United States). After blocking with 5% non-fat milk for 2 h, the membranes were incubated with primary antibodies against HuR (1:1000), IL-1 (1:800), TNF-α (1:800), p65 (1:1000), MMP3 (1:1000), MMP13 (1:1000), IL-6 (1:800), Aggrecan (1:1000), COL2 (1:1000), NKRF (1:1000), p-p65 (1:800), IkBa (1:800), Lamin B (1:2000), and GAPDH (1:3000) overnight at 4°C, and then subsequently incubated with the respective secondary antibodies for 2 h at room temperature. After washing three times with Tris-buffered saline with Tween^®^ 20, the blots were visualized by electrochemiluminescence plus reagent (Invitrogen). The signals were visualized using the ChemiDoc^TM^ XRS + Imaging System (Bio-Rad, Hercules, CA, United States), and the band densities were quantified with Image Lab 3.0 software (Bio-Rad, Hercules, CA, United States).

### Immunofluorescence

Rat NP cells were plated in a six-well plate and treated as described above. After treatment, the NP cells were washed with ice-cold PBS, fixed with 4% (v/v) paraformaldehyde for 15 min, and permeated using 0.1% Triton X-100 diluted in PBS for 10 min. Then, the cells were blocked with 5% bovine serum albumin for 1 h at 37°C and then incubated with a primary antibody at 4°C overnight. The next day, the cells were incubated with Alexa Fluor^®^ 488- or Alexa Fluor^®^ 594-conjugated secondary antibodies (1:300) for 1 h at room temperature and labeled with DAPI for 5 min. Finally, five fields from each slide were chosen randomly for microscopic observation with a Nikon ECLIPSE Ti microscope (Nikon, Japan), and the fluorescence intensity was measured using ImageJ software 2.1 (Bethesda, MD, United States) by observers who were blinded to the experimental groups.

### Lentivirus and siRNA Transfection

The NP cells reaching 40–60% confluence were transfected using LV-siHuR lentivirus (from Cell-land, China, TACCAGTTTCAATGGTCATAA), at a multiplicity of infection (MOI) of 30. The NP cells reaching 40–60% confluence was transfected using LV-HuR lentivirus (from OBiO, China, NM_001108848), at a MOI of 50. The NP cells reaching 40–60% confluence were transfected using LV-NKRF lentivirus (from GeneChem, China, XM_003752133), at a MOI of 40. After 12 h of transfection, the culture medium was changed every other day. When confluent, the transfected NP cells were passaged for further experiments.

### RIP

RNA-binding protein immunoprecipitation (RIP) can be used to identify specific RNA molecules, which are associated with targeted proteins. The immunoprecipitation of endogenous ribonucleoprotein complexes was analyzed using Imprint^®^ RNA Immunoprecipitation Kit (Sigma) according to the manufacturer’s instructions.

### Puncture-Induced Rat IVDD Model

Adult male Sprague-Dawley rats (200–250 g, *n* = 32) used for this study were randomly obtained from the Experimental Animal Institute of Wenzhou Medical University and were randomly divided into four groups: Control group, IVDD group, IVDD + LV-NKRF group, and IVDD + LV-HuR group. The experimental level rat tail disc (Co7/8) was located by digital palpation on the coccygeal vertebrae and confirmed by counting the vertebrae from the sacral region in a trial radiograph. The tail skin was sterilized with iodinated polyvinylpyrrolidone, and needles (27G) were used to puncture the whole layer of AF through the tail skin. To make sure the needle is not be punctured too deep, the length of the needle was decided according to the AF and NP dimensions, which were measured in the preliminary experiment about 5 mm. Subsequently, 5 μl of lentivirus (virus type: LV-NC, LV-NKRF, or LV-HuR; virus concentration: 10^9^ TU/ml) was injected into the NP cavity by using a microliter syringe through that 27 G needle (Zheng et al., 2019; Chen Y. et al., 2020). All animals were allowed free unrestricted weight bearing and activity and were monitored every day to ensure their well-being.

### X-Ray Image Acquisition and Magnetic Resonance Imaging (MRI)

Four weeks after surgery, X-ray images were obtained for all animals. The rats were fixed in a prone position in the X-ray irradiation system (Kubtec Medical Imaging, United States). The disc height index (DHI) was determined using the published method.

The rats’ coccyx was analyzed in sagittal T2-weighted images using a 3.0-T clinical magnet (Philips Intera Achieva 3.0 MR). T2-weighted sections in the sagittal plane were obtained with the previous settings (Zheng et al., 2019). The magnetic resonance imagings (MRIs) were evaluated by a blinded orthopedic researcher using the Pfirrmann MRI grading system (Pfirrmann et al., 2001).

### Histopathological Analysis and Immunohistochemical Examination

The rats were killed by an intraperitoneal overdose of 4% pentobarbital, and the tails were collected. The specimens were decalcified, fixed in formaldehyde, dehydrated, and embedded in paraffin. Then, the tissues were cut into 5-μm sections. Slides of each disc were stained with hematoxylin and eosin (H&E) and Safranin O-fast green (S-O). The cellularity and morphology of the intervertebral disc were examined by a separate group of experienced histological researchers in a blinded manner using a microscope (Olympus Inc., Tokyo, Japan).

After deparaffinization, sections of sample for immunohistochemistry were incubated with 3% H_2_O_2_ for 10 min and washed by PBS for three times. Then, the sections were incubated with 0.1% trypsin for 20 min and washed by PBS for three times. Sections were blocked with 1% (w/v) goat serum albumin for 1 h at 37°C, followed by primary antibody (1:200) incubation at 4°C overnight. Negative control sections were incubated with non-specific IgG. Next, the sections were washed three times with PBS and incubated with HRP-conjugated secondary antibodies for 1 h at 37°C. At least three sections from each specimen were observed. The rate of positive cells each section was quantitated by observers who were blinded to the experimental groups.

### Statistical Analysis

Data are presented as the means ± standard deviation (SD) from three independent experiments. The data were analyzed via GraphPad Prism (United States). Statistical differences between two groups were determined using two-tailed unpaired Student’s *t* test, or two-tailed non-parametric Mann–Whitney test. In multiple comparisons, multiple groups were performed with one-way analysis of variance (ANOVA) and Tukey’s *post hoc* test. *p* values < 0.05 were considered statistically significant.

## Results

### The Expression of HuR Is Decreased in Degenerative NP Tissues and TNF-α-Treated NP Cells

**FIGURE 1:**
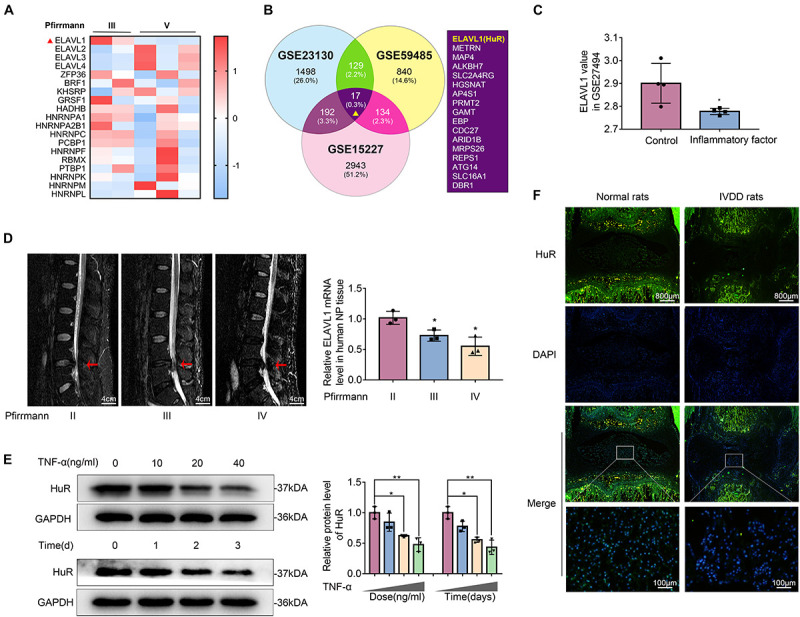
The expression of HuR is decreased in degenerative NP tissues and TNF-α-treated NP cells. **(A)** The heatmap, from GSE23130, displayed the expressions of 19 known post-transcriptional genes. **(B)** Venn diagram of all differentially downregulated genes (*p* < 0.05, FC < 0) and data from different NP tissues (GSE23130, GSE59485, and GSE15227). **(C)** The ELAVL1 gene expression from GSE27494 in NP cells under inflammatory factor stimulation. **(D)** Representative MRI images of three different degrees of IVDD patients, and the ElAVL1 gene expression of NP tissue (The red arrow indicates the intervertebral disc segment used in the experiment). **(E)** The ELAVL1 protein expression was detected by Western blot in various concentrations or time of TNF-α-treated NP cells. **(F)** Representative immunofluorescence staining of HuR in NP tissue from normal and IVDD rats (scale bar: 800 μm). All data were shown as mean ± SD. *p < 0.05, **p < 0.01.

Post-transcriptional regulation is the control of gene expression at the RNA level, which contributes substantially to gene expression regulation across tissues. In order to explore the post-transcriptional regulators that are involved in the process of IVDD, we screened data from the GEO DataSets^1^. A typical set of data (Series Accession: GSE23130) was shown as heatmap in Figure 1A. From the heatmap, it was implied that ELAVL1(HuR) was the most differentially expressed post-transcriptional regulator during IVDD process. To further verify the results, two more datasets (GSE59485 and GSE15227) were included. Venn diagram showed that ELAVL1(HuR) was one of commonly downregulated genes during IVDD (Figure 1B). Interestingly, it was found from dataset GSE27494 that ELAVL1(HuR) gene level was also depressed in NP cells under inflammatory factor stimulation (Figure 1C). In short, the GEO DataSets data showed that ELAVL1(HuR) expression is decreased in degenerative NP tissues and NP cells under inflammatory factor stimulation.

In order to further certify that ELAVL1(HuR) expression is decreased in IVDD, we collected NP tissues from IVDD patients (Pfirrmann grade II, III, and IV) to detect HuR expression by qPCR (Figure 1D). The qPCR results showed that ELAVL1(HuR) expression was decreased as the disc degeneration grade rises (Figure 1D). In addition, we established a puncture-induced rat IVDD model to detect HuR expression by immunofluorescence, and the results showed that HuR expression was also decreased in rat degenerative NP tissues (Figure 1F). In short, these results showed that HuR expression was decreased in human degenerative NP tissues and rat degenerative NP tissues.

Furthermore, we isolated NP cells from normal human NP tissues and treated them with ascending concentrations and duration of TNF-α. The results showed that HuR expression was decreased in a time- and dose-dependent manner (Figure 1E).

### HuR Regulates Inflammation in TNF-α-Treated NP Cells

To explore the effects of HuR on IVDD *in vitro*, we regulated HuR expression in NP cells by transfecting with lentivirus-shHuR (LV-shHuR) and lentivirus-HuR (LV-HuR), and the lentivirus transfection efficiency was confirmed by PCR and Western blot (WB) (Supplementary Figures S2A,B). Although there are two reacting bands of HuR in HuR-overexpressing cells, it may not affect the subsequent experiments; similar phenomenon could be found in previous studies (Katsanou et al., 2005; Kawagishi et al., 2013).

**FIGURE 2:**
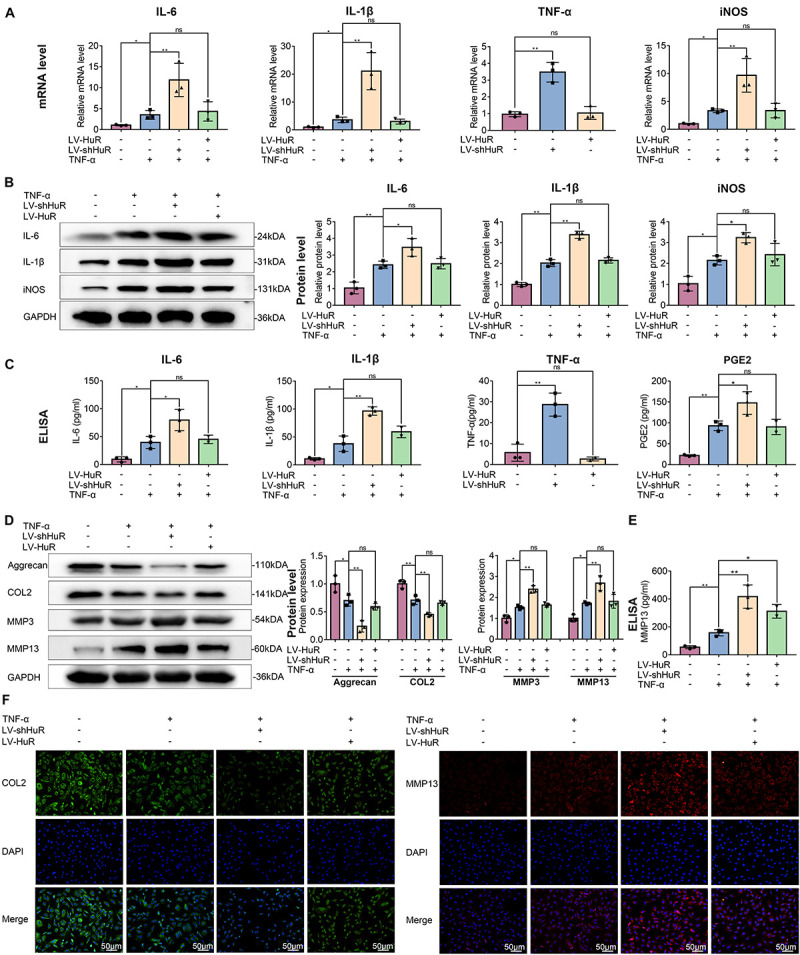
HuR regulates inflammation and ECM degradation in TNF-α-treated NP cells. The cells were transfected with LV-NC, LV-shHuR, or LV-HuR before TNF-α treatment. **(A)** The gene expression of inflammatory cytokines, such as IL-6, IL-1β, TNF-α, and iNOS, was detected, with or without TNF-α treatment and added lentivirus. **(B,C)** The protein expressions of inflammatory cytokines were detected by Western blot and ELISA. **(D)** The protein expressions of ECM biosynthesis and ECM breakdown proteins, such as Aggrecan, COL2, MMP3, and MMP13, were detected by Western blot. **(E)** The expressions of MMP13 detected by ELISA. **(F)** The COL2 and MMP13 were detected by immunofluorescence combined with DAPI staining for nuclei (scale bar: 50 μm). All data were shown as mean ± SD (*n* = 3). *p < 0.05, **p < 0.01.

Next, we detected the expressions of IL-6, IL-1β, TNF-α, and iNOS (by qPCR, WB, and ELISA), which are commonly used as indicators of inflammation, in NP cells. The qPCR results showed that the expression of IL-6, IL-1β, TNF-α, and iNOS were expectedly increased under TNF-α treatment; interestingly, proinflammatory mediators were higher in the HuR-deficient and TNF-α-treated group (LV-shHuR + TNF-α) than those in the TNF-α-treated alone group (Figure 2A). Meanwhile, the WB and ELISA results also confirmed the qPCR results (Figures 2B,C). These results indicate that downregulation of HuR promotes inflammation in TNF-α-treated NP cells.

Downregulation of HuR promotes inflammatory response in TNF-α-treated NP cells; however, to our surprise, HuR upregulation could not inhibit inflammation in TNF-α-treated NP cells. The results of qPCR, WB, and ELISA showed that the expressions of IL-6, IL-1β, TNF-α, and iNOS in HuR overexpression and the TNF-α-treated group (LV-HuR + TNF-α) were not lower than those in the TNF-α group (Figure 2).

### HuR Regulates ECM Metabolism in TNF-α-Treated NP Cells

Extracellular matrix degradation, induced by excessive catabolism and inadequate anabolism, is considered to be one of the most prominent feature in NP cells during IVDD (Hua et al., 2017). We then analyzed Aggrecan and collagen II (COL2) expression to determine ECM anabolism and the expression of MMP3 and MMP13 to determine ECM catabolism in HuR-overexpressing and knockdown NP cells. The qPCR and Western blotting results showed that TNF-α treatment significantly reduced the expression of Aggrecan and COL2, while it increased the expressions of MMP3 and MMP13 (Figure 2D and Supplementary Figure S3); interestingly, compared with the TNF-α treatment alone group, the expressions of Aggrecan and COL2 were decreased, while the expressions of MMP3 and MMP13 were further increased in the TNF-α + LV-shHuR group. Meanwhile, immunofluorescence and ELISA analyses of COL2 and MMP13 also confirmed the phenomenon (Figures 2D,F). To our surprise, HuR upregulation could not maintain ECM homeostasis in the TNF-α-treated NP cell (Figure 2).

### HuR Deficiency Activates NF-κB Signaling Pathway in TNF-α-Treated NP Cells

**FIGURE 3:**
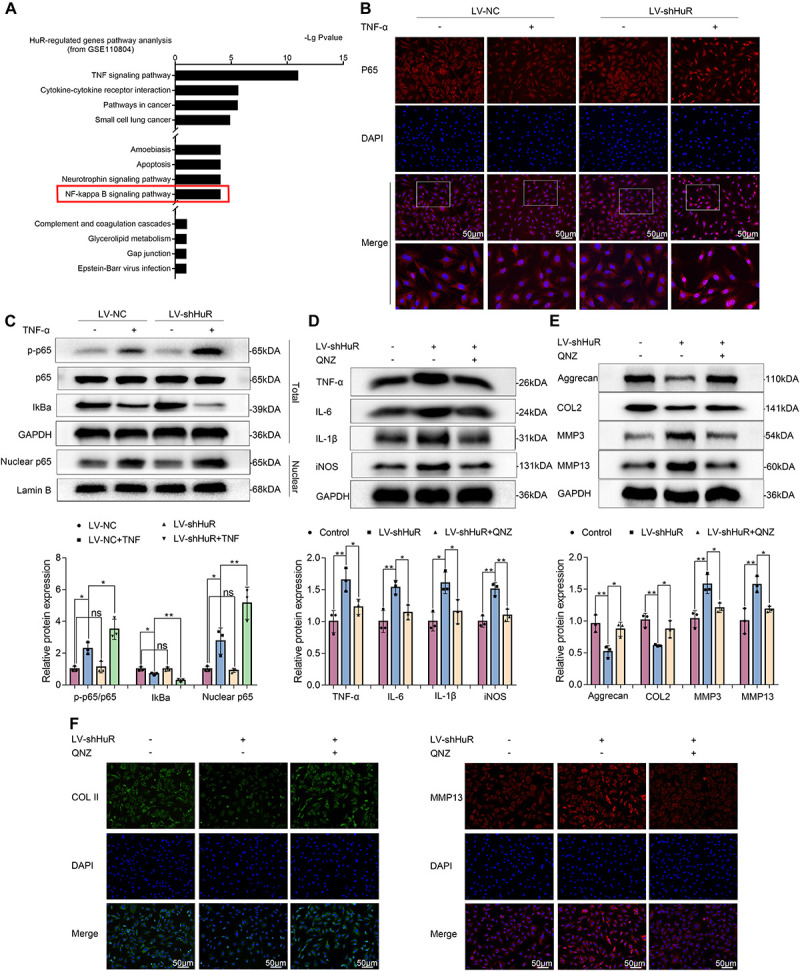
HuR regulates NF-κB signaling pathway in TNF-α-treated NP cells. The cells were transfected with LV-shHuR before TNF-α treatment. **(A)** The KEGG pathway analysis included HuR-regulated genes (top 1000), from GSE110804, and the regulation of NF-κB signaling pathway was put forward. **(B)** The p65 was detected by immunofluorescence combined with DAPI staining for the nuclei (scale bar: 50 μm). **(C)** The protein expression of p-p65, p65, IκBα, and nucleus p65 was detected by Western blot in the NPCs, with or without TNF-α treatment and added lentivirus. **(D)** The protein expression of IL-6, IL-1β, TNF-α, and iNOS was detected by Western blot, with or without LV-shHuR lentivirus and added NF-κB signaling pathway inhibitor QNZ. **(E)** The protein expression of Aggrecan, COL2, MMP3, and MMP13 was detected by Western blot. **(F)** The COL2 and MMP13 were detected by immunofluorescence combined with DAPI staining for nuclei (scale bar: 50 μm). All data were shown as mean ± SD (*n* = 3). *p < 0.05, **p < 0.01.

In order to explore the working mechanism of HuR on IVDD in NP cells, we analyzed genes that have been reported to be regulated by HuR in previous studies. The study by Mukherjee et al. (2011) was included into KEGG pathway analysis. The results showed that various pathways were involved, among which NF-κB signaling pathway is most likely to participate in the regulatory effects of HuR on inflammation in IVDD (Figure 3A). The NF-κB signaling pathway is a key regulator of inflammation (Liu T. et al., 2017); and evidences demonstrated that inhibition of NF-κB signaling pathway can delay the progression of IVDD (Liu T. et al., 2017; Chen F. et al., 2020). Thus, we focus on the NF-κB signaling pathway in further studies.

We evaluated the NF-κB signaling pathway by WB, and the results showed that the NF-κB signaling pathway was dramatically activated in TNF-α-treated NP cells, as indicated by the p-p65/p65 ratio, the IκBα expression in total cell, and the p65 expression in nuclear (Figure 3C); interestingly, the NF-κB signaling pathway was further activated in the LV-shHuR + TNF-α group (Figure 3C). These results were also confirmed by immunofluorescence (Figure 3B).

To determine whether inhibiting NF-κB signaling pathway may suppress inflammation and ECM degradation caused by HuR deficiency, NP cells were pre-treated with the classical NF-κB inhibitor QNZ (EVP4593). The WB results showed that there were significantly higher levels of inflammation and ECM degradation in HuR-deficient NP cells, while this phenomenon was attenuated by QNZ treatment (Figures 3D,E). The ECM results were also confirmed by immunofluorescence (Figure 3F).

### HuR Regulates NF-κB Signaling Pathway Through NKRF in TNF-α-Treated NP Cells

**FIGURE 4:**
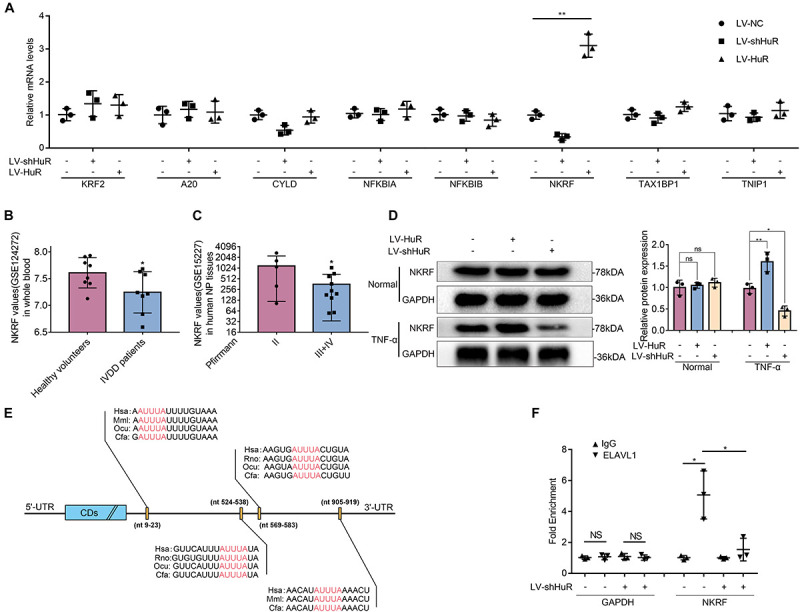
HuR regulates NF-κB signaling pathway through NKRF in TNF-α-treated NP cells. The cells were transfected with LV-shHuR or LV-HuR before TNF-α treatment. **(A)** The known proteins that inhibit NF-κB activation were listed, and the multiple mRNA expressions were detected by qPCR after TNF-α treatment. **(B)** The NKRF gene expression from GSE124272 in human whole blood. **(C)** The NKRF gene expression from GSE15227 in human degenerated NP tissues. **(D)** The protein expression of NKRF was detected by Western blot, treated with or without TNF-α treatment; the levels were determined using Image J software. **(E)** The potential HuR-binding element in 3′UTR of NKRF mRNA on multiple species. **(F)** The mRNA expression of NKRF was detected by qPCR after RIP in rat NP cells, normalized to IgG isotype controls. All data were shown as mean ± SD (*n* = 3) *p < 0.05, **p < 0.01.

Next, we asked through which protein does HuR exert its inhibitory effect on NF-κB signaling pathway. We tested known proteins that may inhibit NF-κB activation by qPCR, which include KLF2 (Jha and Das, 2017), A20 (Wertz et al., 2004), Cyld (Trompouki et al., 2003), NFKBIA (Rinkenbaugh and Baldwin, 2011), NFKBIB (Chapman et al., 2007), NKRF (Nourbakhsh and Hauser, 1999), Tax1bp1 (Shembade et al., 2011), and Tnip1 (Huang et al., 2008). The results showed that the expression of NKRF was the most significantly affected by HuR expression (Figure 4A).

The data from the GEO DataSets also showed that the expression of NKRF was decreased in both blood and intervertebral disc tissues of IVDD patients (Figures 4B,C). Meanwhile, we evaluated the expression of NKRF under TNF-α treatment condition in NP cells by WB; it was shown that the expression of NKRF was increased in HuR-overexpressing NP cells, whereas it decreased in HuR knockdown NP cells. We also assessed the expression of NKRF in NP cells without TNF-α treatment; it showed that NKRF was not affected by HuR expression in TNF-α untreated NP cells (Figure 4D).

Previous studies have shown that HuR may exert its effect on gene expression through stabilizing mRNAs in a variety of biological processes (Lopez De Silanes et al., 2004). Bioinformatic study implied that there are multiple HuR binding sites at the 3′UTRs of NKRF mRNA (Figure 4E). In the following study, we verified the binding between HuR and NKRF mRNA by RIP experiments. mRNAs were evaluated by qPCR in HuR pull-down molecules. The results showed that the expression of NKRF mRNA is highly expressed in HuR pull-down molecules; however, when HuR was knocked down, the expression of NKRF mRNA was significantly decreased (Figure 4F), suggesting that HuR may indeed bind to NKRF mRNA.

### NKRF Suppresses NF-κB Signaling Pathway in HuR-Deficient NP Cells and Suppresses Inflammation and ECM Degradation in TNF-α-Treated NP Cells

**FIGURE 5:**
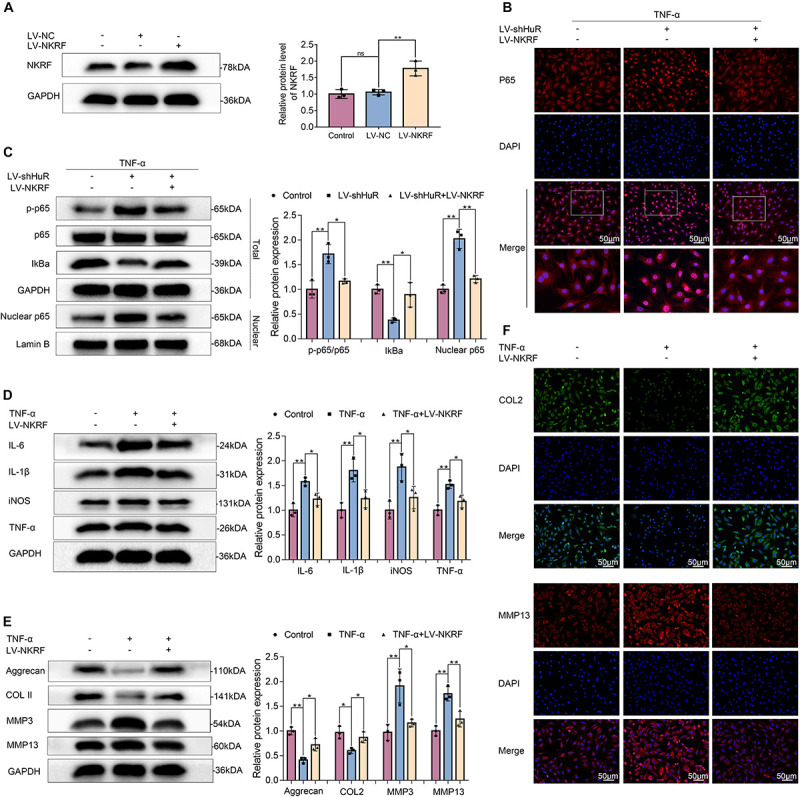
NKRF regulates NF-κB signaling pathway, inflammation, and ECM degradation in NP cells. The cells were transfected with LV-shHuR or LV-NKRF before TNF-α treatment. **(A)** The protein expression of NKRF was detected by Western blot in rat NP cells; the levels were determined using Image J software. **(B)** The p65 was detected by immunofluorescence combined with DAPI staining for nuclei (scale bar: 50 μm). **(C)** The protein expression of p-p65, p65, IκBα, and nucleus p65 was detected by Western blot in the NPCs, with or without added lentivirus. **(D)** The protein expression of IL-6, IL-1β, TNF-α, and iNOS was detected by Western blot, with or without LV-NKRF lentivirus and TNF-α. **(E)** The protein expression of ECM biosynthesis and ECM breakdown proteins, such as Aggrecan, COL2, MMP3, and MMP13, with or without LV-NKRF lentivirus and TNF-α. **(F)** COL2 and MMP13 expressions were detected by immunofluorescence combined with DAPI staining for nuclei (scale bar: 50 μm). All data were shown as mean ± SD (*n* = 3) *p < 0.05, **p < 0.01.

NKRF may regulate NF-κB signaling pathway under physiological conditions; however, whether it may regulate NF-κB signaling pathway under HuR-deficient conditions is still unknown. Thus, we upregulated NKRF expression in HuR-deficient NP cells by transfecting them with lentivirus-NKRF (LV-NKRF). The WB results showed that the NKRF levels increased by LV-NKRF transfection, implying that we have successfully established the NKRF upregulation NP cell model (Figure 5A). Next, we assessed whether upregulating NKRF may inhibit NF-κB signaling pathway induced by HuR overexpression. The WB results showed that the NF-κB signaling pathway was dramatically activated in the LV-shHuR group, as indicated by the p-p65/p65 ratio, the IκBα expression in total cell, and the p65 expression in nuclear, whereas NKRF upregulation can inhibit NF-κB signaling pathway (Figures 5B,C).

To determine the function of NKRF during the IVDD process, NKRF was overexpressed in TNF-α-treated NP cells, and inflammation and ECM degradation were assessed. The WB results showed that the increased levels of TNF-α, IL-6, IL-1β, and iNOS induced by TNF-α treatment were attenuated by NKRF overexpression (Figure 5D); the degradation of ECM induced by TNF-α was also attenuated by NKRF overexpression (Figures 5E,F).

### Overexpression of NKRF, but Not HuR, Ameliorates the Process of IVDD *in vivo*

To investigate the protective effects of HuR and NKRF in IVDD *in vivo*, we established puncture-induced rat IVDD model and injected lentivirus into the intervertebral disc to regulate the expression of HuR and NKRF.

**FIGURE 6:**
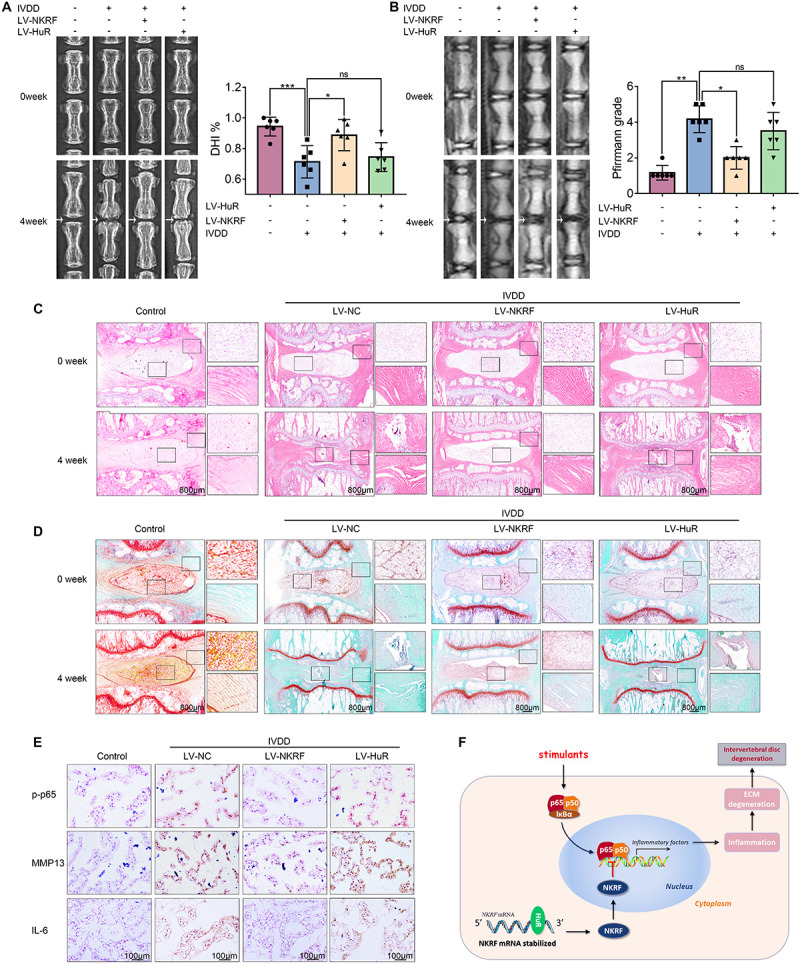
NKRF, but not HuR, overexpression ameliorates the process of IVDD *in vivo*. The rats were injected lentivirus into the intervertebral disc. **(A)** The X-ray of a rat-tail disc at 4 weeks after disc puncture surgery in normal and IVDD rats, with or without lentivirus transfection (white arrows); the disc height index (DHI) of a rat-tail disc. **(B)** T2-weighted MRI of a rat-tail disc at 4 weeks after disc puncture surgery (white arrows); The respective Pfirrmann grade scores of a rat-tail disc. **(C,D)** Representative hematoxylin and eosin staining and Safranin O-Fast Green staining of NP tissues in normal and IVDD rats (bar: 800 μm). **(E)** The immunohistochemical staining of p-p65, MMP13, and IL-6 in intervertebral disc sections (bar: 100 μm). **(F)** Schematic illustration of the research. NKRF inhibits inflammation and ECM degradation in IVDD *via* NF-κB signaling pathway; mechanism study revealed that HuR prompts NKRF mRNA stability via binding to the AU-rich element. All data were shown as mean ± SD. *p < 0.05, **p < 0.01, ***p < 0.001.

The X-ray results showed that disc height was markedly decreased in the IVDD group; however, the disc height was higher in the LV-NKRF group compared to the IVDD group, whereas there was no significant difference between the LV-HuR group and the IVDD group (Figure 6A). Meanwhile, we observed that the T2-weighted signal intensity of the intervertebral disc in the LV-NKRF group was brighter than the one in the IVDD group, whereas the MRI T2-weighted signal intensity in the LV-HuR group was as weak as that in the IVDD group (Figure 6B). Pfirrmann grade scores were also determined. The scores were lower in the LV-NKRF group, and no significant difference was found in the LV-HuR group compared with the IVDD group (Figure 6B).

From H&E staining, we found that the NP tissues were reduced and replaced by the fibrochondrocytes, and the cartilaginous endplate was collapsed in the IVDD group, whereas NKRF upregulation ameliorates these degenerations (Figure 6C). According to Safranin O-Fast Green staining, which is sensitive to proteoglycan and hyaluronic acid, we also found that red staining signal in the LV-NKRF group was higher than those in the IVDD group, whereas red staining signal in NP tissues in the LV-HuR group was similar to that in the IVDD group (Figure 6D).

Further, the results showed that the inflammation and ECM degradation were prompted in the IVDD group, as indicated by the expression of MMP13 and IL-6 (detected by immunohistochemistry, Figure 6E), whereas NKRF overexpression ameliorated the increased inflammation and ECM degradation. In order to investigate the regulatory effect of NKRF on NF-κB signaling pathway *in vivo*, we detected the expression of p-p65 expressions by immunohistochemical staining. The results showed that the levels of p-p65 expressions were prompted in the IVDD group, whereas NKRF upregulation inhibited the expression of p-p65 expressions (Figure 6E). Unfortunately, we also found that HuR overexpression cannot inhibit the expression of MMP13, IL-6, and p-p65, indicating that HuR overexpression may not ameliorate inflammation, ECM degradation, and NF-κB signaling pathway *in vivo*.

## Discussion

Intervertebral disc degeneration has been reported to be a major cause of low back pain. In the current study, we found that HuR may suppress inflammatory response and maintain ECM homeostasis in NP cells *via* inhibiting NF-κB signaling pathway *in vitro*; a mechanism study revealed that HuR prompts NKRF mRNA stability *via* binding to the AU-rich element, and upregulation of NKRF inhibits NF-κB signaling pathway and inflammatory response and maintains ECM homeostasis in HuR-deficient NP cells. *In vivo*, we demonstrated that NKRF overexpression ameliorates the process of IVDD (Figure 6F).

Human antigen R is an RNA-binding protein, and HuR stabilizes mRNAs to regulate gene expression, which in turn affects various and contradictory biological processes (Peng et al., 1998; Zhang et al., 2018). Here, we asked whether HuR plays a protective or destructive role in IVDD. Shang et al. (2015) found that HuR promotes the progression of diabetic nephropathy and regulates NADPH oxidase, by regulating the mRNA of NOD2. Zhang and Bowden (2008) found that HuR upregulation promotes non-melanoma skin cancer under UVB by maintaining the stability of COX-2 mRNA. On the other hand, Liu L. et al. (2017) found that HuR upregulation enhances the early recovery of the intestinal epithelium after acute injury via increasing the translation of Cdc42. In this study, we found that the role of HuR in IVDD is complicated; although its overexpression may not alleviate IVDD pathology, its knockdown may exacerbate IVDD process in TNF-α-treated NP cells.

Inflammatory factors play a crucial role in the development of IVDD. However, the regulation of HuR in inflammation is controversial. Mubaid et al. (2019) found that HuR overexpression promotes the inflammation by promoting the translation of STAT3 in muscular dystrophy, and Krishnamurthy et al. (2010) found that HuR knockdown can attenuate the inflammatory response after myocardial infarction in IL-10-deficient mice, by reducing the mRNA expression of TNF-α and TGF-β; however, Yiakouvaki et al. (2012) found that high expression of HuR can protect mice from inflammation in pathological enteritis; Ceolotto et al. (2014) found that HuR suppresses chronic inflammation, associated with endothelial injury, by stabilizing the mRNA of SIRT1. In the current study, we found that HuR is negatively related to inflammation in IVDD, because the expression of IL-6, IL-1β, TNF-α, and iNOS was higher in HuR knockdown and the TNF-α-treated group than in the TNF-α-alone group. At the same time, we found that knockdown of HuR inhibits ECM anabolism (Aggrecan, COL2) and promotes ECM catabolism (MMP3, MMP13).

The NF-κB signaling pathway plays a significant role in inflammation (Liu T. et al., 2017; Ke et al., 2019). Studies have reported that abnormal activation of the NF-κB signaling pathway is closely related to the occurrence and development of IVDD (Wuertz et al., 2012), and inhibition of the NF-κB signaling pathway can delay the progression of IVDD (Liu T. et al., 2017; Taniguchi and Karin, 2018). At the same time, the KEGG pathway analysis of the HuR-regulated gene chip suggests that the NF-κB signaling pathway plays a role under HuR regulation. However, the regulatory role of HuR on the NF-κB signaling pathway is controversial. Some studies have shown that HuR activates the NF-κB signaling pathway. Liu and Yu (2014) found that HuR overexpression in 3T3 cells promotes the expression of IκBα protein and activates the NF-κB signaling pathway under heat shock treatment, and Rhee et al. (2010) found that knockdown of HuR with siRNA can inhibit the phosphorylation of NF-κB in endothelial cells to inhibit the NF-κB signaling pathway. However, some studies have also shown that HuR may suppress the NF-κB pathway. Ceolotto et al. (2014) found that in chronic inflammation, HuR inhibits the NF-κB signaling pathway by stabilizing SIRT1 mRNA, while Hashimoto et al. (2014) found in fibroblasts that the lack of HuR activates the NF-κB signaling pathway. In our study, the results showed that under TNF-α stimulation conditions, HuR knockdown may promote NF-κB signaling pathway activation, while NF-κB pathway inhibitor may alleviate the inflammation induced by HuR knockdown.

Next, we explored the target molecule through which HuR exerts its suppressive effects on NF-κB signaling pathway. We tested known proteins that inhibit NF-κB activation and found that the expression of NKRF has the most significant difference. The data in NCBI also showed that the expression of NKRF decreased in both blood and NP tissues of IVDD patients, and the WB results of this study also showed that the expression of NKRF was regulated by the HuR. Furthermore, we found that there are obvious HuR binding sites at the 3′UTRs of NKRF mRNA, and RIP experiments showed that NKRF mRNA can indeed bind to HuR, and the expression of NKRF mRNA is positively correlated to HuR protein expression. In short, NKRF is likely to be the target of HuR.

NKRF, which is abundant in many cells and tissues, is mainly located in the nucleolus and also in the cytoplasm and nucleoplasm (Niedick et al., 2004). NKRF interacts with specific negative regulatory elements to regulate the transcriptional activity of NF-κB complexes, which inhibits the expression of certain NF-κB response genes (Feng et al., 2002; Akool El et al., 2003; Gealy et al., 2005). In this research, upregulating the expression of NKRF can inhibit the inflammation and ECM degradation induced by HuR knockdown; meanwhile, we found that the NKRF upregulation can directly inhibit the inflammation induced by TNF-α, reflecting the ability and potential of NKRF to regulate inflammation and ECM degradation directly. However, there are also studies that reported a different role of NKRF in inflammation. Froese et al. (2006) demonstrated that when attacked by pathogens, the survival of NKRF gene-deficient mice showed no difference from wild-type mice; it was proposed that NKRF may be a redundant gene. The contradiction suggests that the role of NKRF in inflammation may be stimulation dependent and cell type dependent.

Based on the function of HuR in NP cells, we tried to upregulate HuR to rescue the TNF-α-induced inflammation and ECM degradation. To our surprise, although the downregulation of HuR caused an increase in inflammation and degradation of ECM in the TNF-α-treated NP cells, upregulation of HuR could not inhibit the inflammation and ECM degradation.

One possible reason is that HuR may have a pleiotropic effect by interacting with different mRNAs (Supplementary Figure S4). Gealy et al. (2005) found that HuR promotes the stability of IL-6 mRNA in human fibroblasts when they were infected with human cytomegalovirus; Suzuki et al. (2006) found in rheumatoid arthritis synovium that HuR may promote the stability of TNF-α mRNA; Linker et al. (2005) reported that HuR could promote the expression of iNOS mRNA in human epithelial colon cancer cells. Therefore, although it was shown in our research that HuR may promote NKRF, which, in turn, inhibits the NF-κB signaling pathway and helps NP cells to combat inflammation and ECM degradation, the overexpression of HuR may also directly upregulate mRNA that may promote inflammation and ECM degradation. Thus, we propose that HuR may not serve as a therapeutic target for IVDD. Instead, we verified that NKRF overexpression may suppress inflammation and ECM degradation in TNF-α-treated NP cells and ameliorate the process of IVDD *in vivo*. In short, NKRF, but not HuR, may serve as therapeutic target for IVDD (Figure 6F).

## Conclusion

In the current study, we demonstrate that the expression of HuR is decreased in NP cells during the process of IVDD, and downregulation of HuR promotes IVDD *via* activating NF-κB signaling pathway; a mechanism study reveals that HuR prompts NKRF mRNA stability *via* binding to the AU-rich element. HuR upregulation could not ameliorate the process of IVDD, while upregulation of NKRF inhibits NF-κB signaling pathway and inflammatory response and maintains ECM homeostasis in HuR-deficient NP cells. Thus, overexpression of NKRF, not HuR, ameliorates the process of IVDD (Figure 6F).

## Data Availability Statement

The original contributions presented in the study are included in the article/Supplementary Material, further inquiries can be directed to the corresponding author/s.

## Ethics Statement

The studies involving human participants were reviewed and approved by the Second Affiliated Hospital and Yuying Children’s Hospital of Wenzhou Medical University Ethics Committee. The patients/participants provided their written informed consent to participate in this study. The animal study was reviewed and approved by the Animal Care and Use Committee of Wenzhou Medical University.

## Author Contributions

XW, XZ, and YFZ designed the experiments. ZS, YS, and LC performed the experiments. ZT and SL wrote the manuscript. AW, YW, and NT analyzed the data and prepared all the figures. LS, ZP, and WG constructed the animal models. All authors reviewed and agreed on the manuscript.

## Conflict of Interest

The authors declare that the research was conducted in the absence of any commercial or financial relationships that could be construed as a potential conflict of interest.

## Footnotes

1 https://www.ncbi.nlm.nih.gov/gds
