# Supplementary material for: RNA-Binding Protein HuR Suppresses Inflammation and Promotes Extracellular Matrix Homeostasis via NKRF in Intervertebral Disc Degeneration
Source: Front Cell Dev Biol. 2020 Nov 25;8:611234. doi: 10.3389/fcell.2020.611234 (PMC7732619; doi:10.3389/fcell.2020.611234)
Supplement: Supplementary file 1 [file Data_Sheet_1.docx]

**Supplementary Figures**


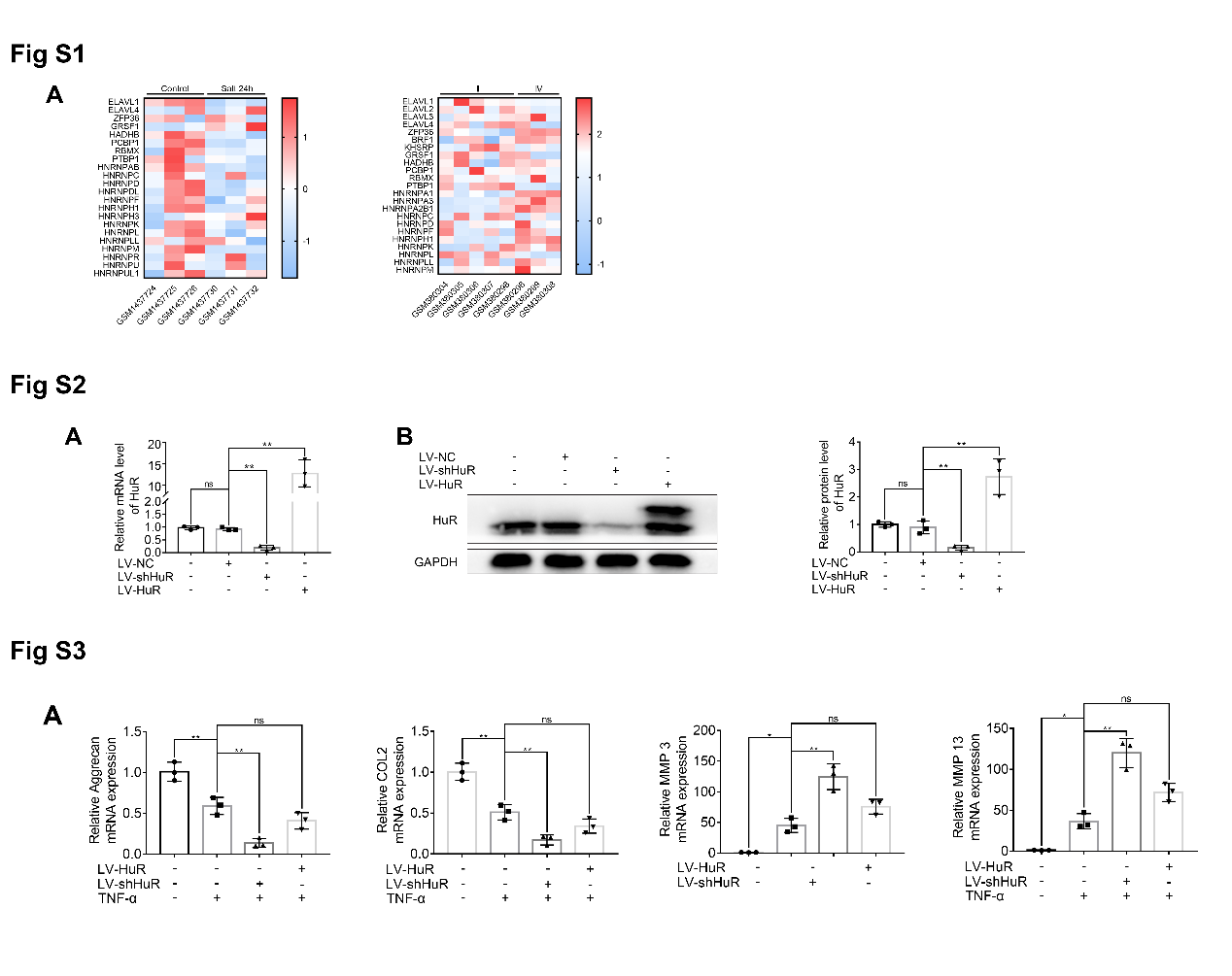


**Supplementary Figure 1.** (A) The heatmaps, from GSE59485 and GSE15227, listed the expressions of known post-transcriptional genes.


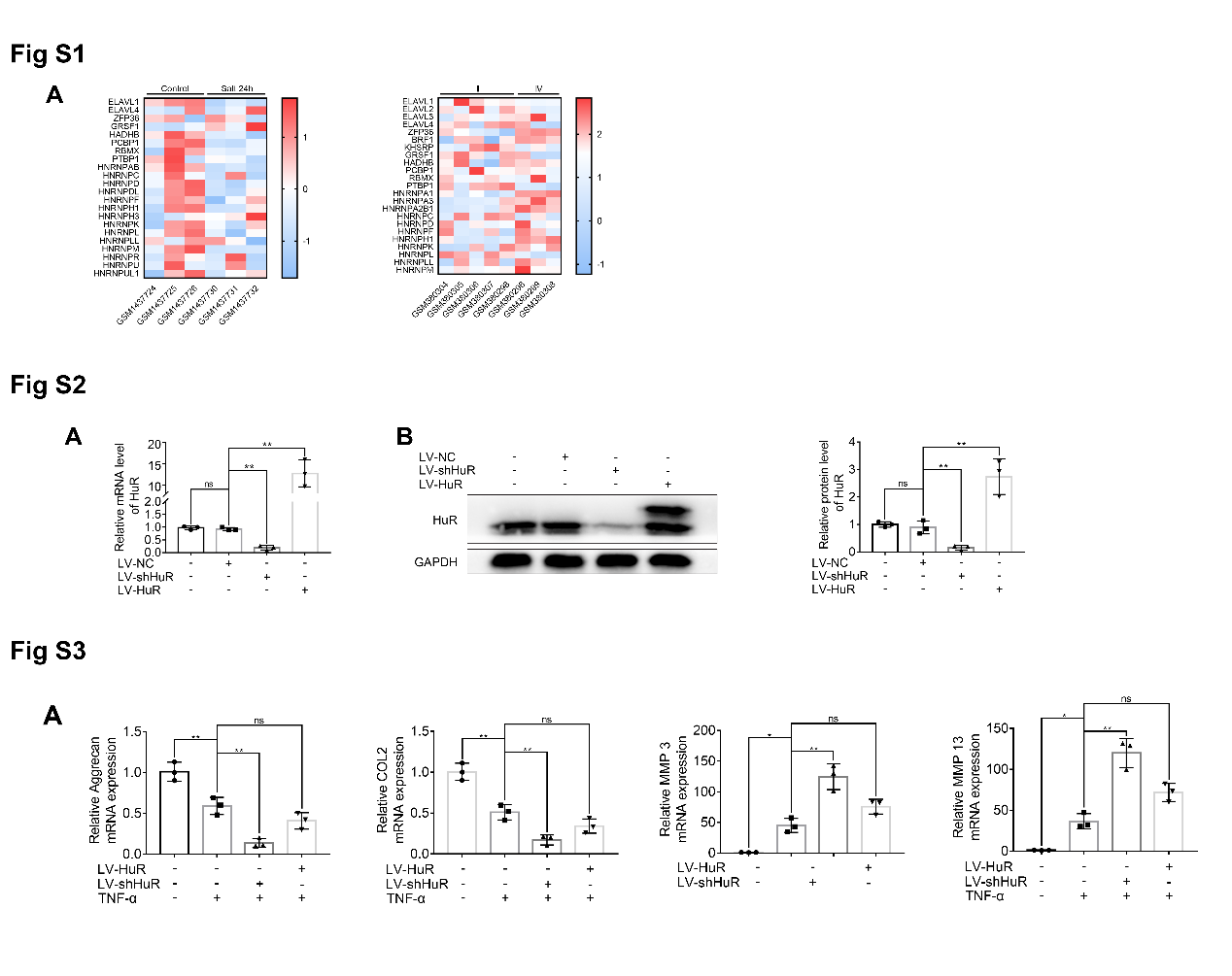


**Supplementary Figure 2.** (A) The ELAVL1 gene expressions were detected by PCR in rat NP cells by transfecting with lentivirus. (B) The protein expressions of HuR were detected by western blot in rat NP cells by transfecting with lentivirus.


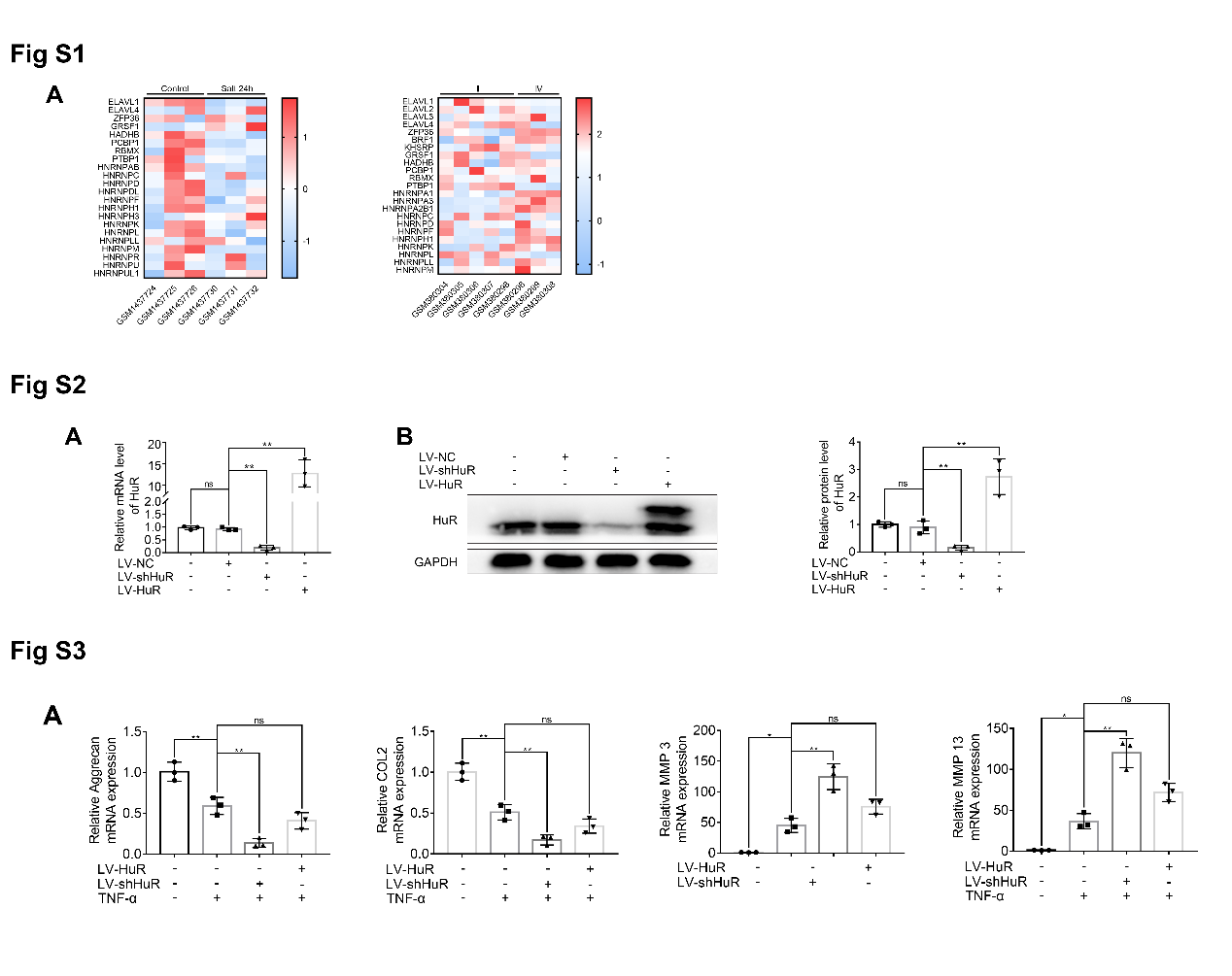


**Supplementary Figure 3.** (A) The gene expression of ECM biosynthesis and ECM breakdown proteins, such as Aggrecan, COL2, MMP3 and MMP13, were detected, with or without TNF-α treatment and added lentivirus.

**Fig S4**


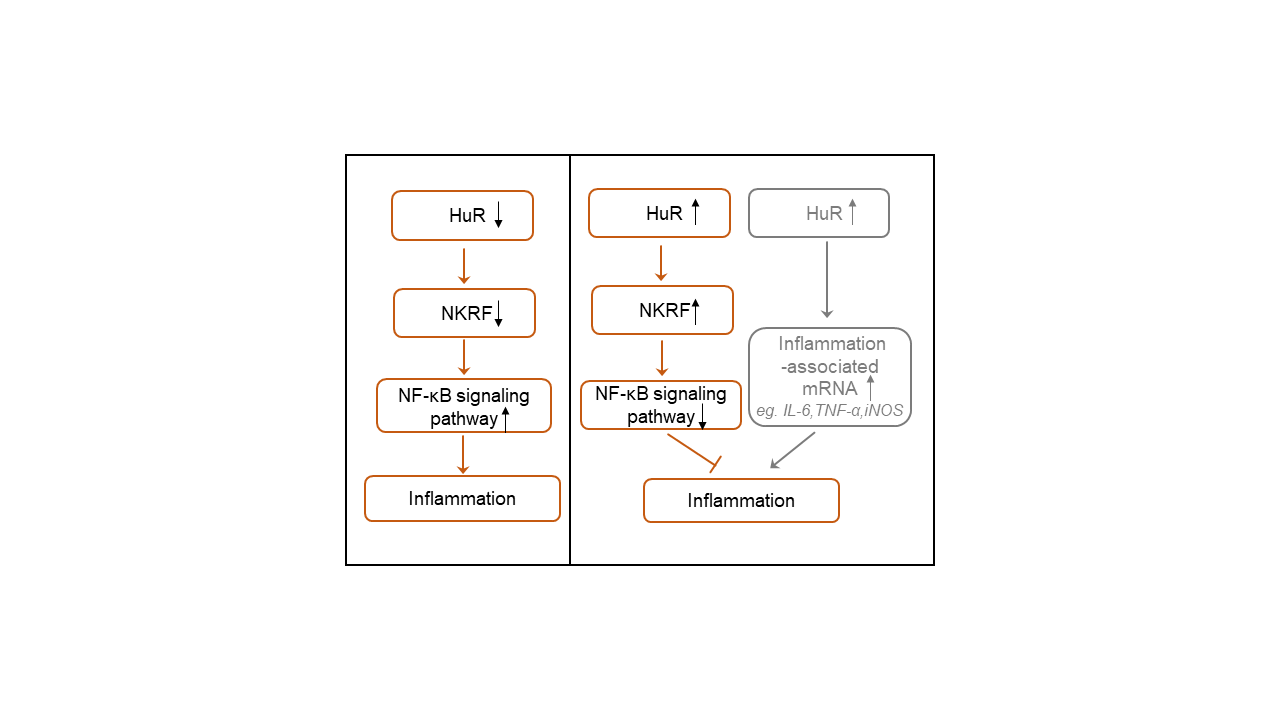


**Supplementary Figure 4.** HuR may have pleiotropic roles on inflammation and ECM degeneration through interacting with different mRNA subsets

When the expression of HuR increased, HuR not only inhibit NF-κB signaling pathway through NKRF to combat inflammation, but also stabilizes inflammation-associated mRNA (*eg.* IL-6, TNF-α, iNOS) to prompt inflammation. Thus, HuR may not be suitable to be a direct target.
