# Supplementary material for: RNA-Binding Protein HuR Suppresses Inflammation and Promotes Extracellular Matrix Homeostasis via NKRF in Intervertebral Disc Degeneration
Source: Front Cell Dev Biol. 2020 Nov 25;8:611234. doi: 10.3389/fcell.2020.611234 (PMC7732619; doi:10.3389/fcell.2020.611234)
Supplement: Supplementary file 2 [file Data_Sheet_2.docx]

**Supplemental Table**

**Primers used for mRNA expression studies using PCR**

| Gene | Forward primer (5'->3') | Reverse primer (3'->5') |
| --- | --- | --- |
| IL-1β | AGGAGAGACAAGCAACGACA | CTTTTCCATCTTCTTCTTTGGGTAT |
| IL-6 | AGTTGCCTTCTTGGGACTGATGT | GGTCTGTTGTGGGTGGTATCCTC |
| TNF-α | GCGTGTTCATCCGTTCTCTACC | TACTTCAGCGTCTCGTGTGTTTCT |
| MMP3 | CTGGAGAATGTGAGTGGG | CTGAAGATGACAGGGAAGC |
| MMP9 | TTGGCTTCCTCCGTGATT | CCCTACTGCTGGTCCTTC |
| MMP13 | AAGCCAAAGAAAGACTGC | CCCCTTCCCTATGGTGAT |
| ADAMTS-4 | GGTTCGGTGGTTGTAGGC | AGGACTTGTGGAGGTGGTG |
| ADAMTS-5 | GTTAGGTGGGCAGGGTAT | GGTCAGTGTTCTCGCTCTT |
| Aggrecan | CAGTGTACTGAGGGCTTTGTCC | GCCTGTGCTTGTAGGTGTTGG |
| iNOS | TCTATCAGGAAGAAATGCAGGAG | ACATCGCCACAAACATAAAGG |
| KLF2 | GAGCCTATCTTGCCGTCCTT | AGCACGCTGTTTAGGTCCTC |
| A20 | ATGGAGTGCCAGCACCTAAG | TTGCCAAAGTGATCGCAAGC |
| CYLD | CCCAGCTCTTCTGGGAATGC | GCTGGCCAATCCAACGGATA |
| NFKBIA | GACGAGGATTACGAGCAGATGGT | GTCTTCTCTTCATGGATGATTGCC |
| Ikbkb | ACTGAGGTGCCCGTGAACTG | TTGTTGTGCTTCTGTGGTGGAG |
| Tax1bp1 | CAGAAAAGGATGAGAAGGAGC | TTTGAGGTTGTAAGCAGGAGA |
| Itch | GGTCGCTGAGTGGAGGTTGTC | TCTGCTTGCTCGTCCTGGTAT |
| Tnip1 | TCCAGAGGGAACGGAGTGAC | ATGGGTGGGTAGGCATAGGG |
| NKRF | GAGGTTAGAGTTGTCCGGCG | GCCATACTTCTGAGCAATCCA |
| GAPDH | TCTCTGCTCCTCCCTGTTC | ACACCGACCTTCACCATCT |
| HuR | GGGCCTCCAAACCTCCTA | GGGCGAATCATCAACTCC |
| DUSP1 | CTGTGCAGCAAACAGTCCAC | AGAGAGGGGTGCTACAGGAG |
| DUSP9 | ACCTTGGACCTGCAAGTTGG | CGCGACGTTGAGAATATGGG |
| DUSP10 | TGTGGCAACCTACGACAAGG | GTGGTCGAGGTTCCTATGGC |
| DUSP11 | AAACCTGCAGAAACGTCGTG | GTCTGAACATGCTGGGACGA |
| RELA | AGACCTGGAGCAAGCCATTAGC | CGGACCGCATTCAAGTCATAGT |
| Nfkb1 | CTATGACAGCAAAGCCCCCAA | CAAATCCTTCCCAAACTCCACC |
